# Development of Harmonized COVID-19 Occupational Questionnaires

**DOI:** 10.1093/annweh/wxac044

**Published:** 2022-07-10

**Authors:** Vivi Schlünssen, Jean Baptist du Prel, Martie van Tongeren, Lode Godderis, Michelle C Turner, Damien McElvenny

**Affiliations:** Department of Public Health, Work, Environment and Health, Danish Ramazzini Centre, Aarhus University, Aarhus C, Denmark; National Research Center for the Working Environment, Copenhagen, Denmark; Department of Occupational Health Science, University of Wuppertal, Wuppertal, Germany; Centre for Occupational and Environmental Health, School or Health Sciences, University of Manchester, Manchester, UK; Centre for Environment and Health, KU Leuven, Leuven, Belgium; IDEWE, External Service for Prevention and Protection at Work, Heverlee, Belgium; Barcelona Institute for Global Health (ISGlobal), Barcelona, Spain; Universitat Pompeu Fabra (UPF), Barcelona, Spain; CIBER Epidemiología y Salud Pública (CIBERESP), Madrid, Spain; Centre for Occupational and Environmental Health, School or Health Sciences, University of Manchester, Manchester, UK; Research Group, Institute of Occupational Medicine, Edinburgh, UK

**Keywords:** COVID-19, occupation, questionnaire, risk factors, SARS-CoV-2

## Abstract

Harmonized tools and approaches for data collection can help to detect similarities and differences within and between countries and support the development, implementation, and assessment of effective and consistent preventive strategies. We developed open source occupational questionnaires on COVID-19 within COVID-19 working groups in the OMEGA-NET COST action (Network on the Coordination and Harmonisation of European Occupational Cohorts, omeganetcohorts.eu), and the EU funded EPHOR project (Exposome project for health and occupational research, ephor-project.eu). We defined domains to be included in order to cover key working life aspects of the COVID-19 pandemic. Where possible, we selected questionnaire items and instruments from existing questionnaire resources. Both a general occupational COVID-19 questionnaire and a specific occupational COVID-19 questionnaire are available. The general occupational COVID-19 questionnaire covers key working life aspects of the COVID-19 pandemic, including the domains: COVID-19 diagnosis and prevention, Health and demographics, Use of personal protective equipment and face covering, Health effects, Work-related effects (e.g. change in work schedule and work–life balance), Financial effects, Work-based risk factors (e.g. physical distancing, contact with COVID-19-infected persons), Psychosocial risk factors, Lifestyle risk factors, and Personal evaluation of the impact of COVID-19. For each domain, additional questions are available. The specific occupational COVID-19 questionnaire focusses on occupational risk factors and mitigating factors for SARS-CoV2 infection and COVID-19 disease and includes questions about the type of job, amount of home working, social distancing, human contact (colleagues, patients, and members of the public), commuting, and use of personal protective equipment and face coverings. The strength of this initiative is the broad working life approach to various important issues related to SARS-CoV-2 infection, COVID-19 disease, and potentially future pandemics. It requires further work to validate the questionnaires, and we welcome collaboration with researchers willing to do this. A limitation is the moderate number of questions for each of the domains in the general questionnaire. Only few questions on general core information like ethnicity, demographics, lifestyle factors, and general health status are included, but the OMEGA-NET questionnaires can be integrated in existing questionnaires about sociodemographic and health-related aspects. The questionnaires are freely accessible from the OMEGA-NET and the EPHOR homepages.

## Background

More than 2 years after the SARS-CoV-2 virus was identified in Wuhan, China, the pandemic still has an immense global health and economic impact on society. By 20 April 2022, there have been an estimated 506 000 000 confirmed cases of COVID-19, and over 6 000 000 deaths (https://covid19.who.int/). The pandemic has initiated a wealth of research on COVID-19 and working life. Examples are research on occupational risk factors for SARS-CoV-2 infection (Beale *et al.*, 2022; Lan *et al.*, 2020; Magnusson *et al.*, 2020; Pearce *et al.*, 2021; Verbeeck *et al.*, 2021), occupational risk factors for COVID-19 disease and mortality (Mutambudzi *et al.*, 2020; Nafilyan *et al.*, 2021; Pearce *et al.*, 2021; Senia *et al.*, 2022), mitigating factors for risks associated with the SARS-CoV-2 virus (Iavicoli *et al.*, 2021; Nelson *et al.*, 2021), and long-term effects of COVID-19 infection often named long-COVID (Pauwels *et al.*, 2021; Rayner and Campbell, 2021). Mental health issues related to being an essential worker during the COVID-19 pandemic has also been studied as reviewed in Froessl and Abdeen (2021).

The majority of occupational research has been conducted among healthcare workers. Despite a strong preventive effort beyond the early stages of the pandemic, healthcare workers still face an increased risk for SARS-CoV-2 infections (Würtz *et al.*, 2022), although studies indicate a decreased risk in second and third wave compared with first wave (Magnusson *et al.*, 2020; Mylle *et al.*, 2021). For other occupations, the knowledge base is still rather limited, and there is an urgent need for occupational COVID-19-related research in order to secure effective preventive measures on the labour market for healthcare workers, other essential workers, and non-essential workers, in particular those who encounter general public contact for the current and future pandemics.

Current research on working life and COVID-19 uses a diversity in approaches, tools, and questionnaires, which makes it difficult to compare data and study results. Moreover, the majority of research has been performed on a national level, despite an urgent need for a global view in order to better understand the nature of the pandemic and the importance of occupational factors. Furthermore, when recording occupational diseases in order to develop preventive measures at the workplace, harmonization of epidemiological surveillance systems and case definitions is of major importance (Van der Molen *et al.*, 2020).

## Open source COVID-19 questionnaires

We aimed to develop open source questionnaires on working life and COVID-19, in order to facilitate the use of harmonized tools and approaches for data collection. This would allow researchers to detect similarities and differences within and between regions and countries. It might facilitate global research on risk and mitigating factors and the impact of adapted preventive measures during the different phases of the pandemic. Furthermore, harmonization of questions is key in order to harmonize and compare epidemiological surveillance systems and case definitions across countries.

The initiative was conducted within COVID-19 working groups in the OMEGA-NET COST action (Network on the Coordination and Harmonisation of European Occupational Cohorts, Omega—OMEGA-NET (omeganetcohorts.eu) (Turner and Mehlum, 2018), and the EU funded EPHOR project (Exposome project for health and occupational research, EPHOR—EPHOR Project (ephor-project.eu) (Pronk *et al.*, 2022).

Initially, we defined domains to be included in order to cover key working life aspects of the COVID-19 pandemic. The domains were selected after two working group meetings in spring and summer 2020 and subsequent discussions. We also screened available tools and questionnaires when the work was initiated in spring 2020, e.g. the repository of COVID tools at the NIOSH Institute of Health (https://www.cdc.gov/niosh/emres/2019_ncov_default.html/), and the UK Household Longitudinal Survey Mainstage Questionnaires (https://www.understandingsociety.ac.uk/sites/default/files/downloads/documentation/mainstage/questionnaire/wave-9/W9-questionnaire-consultation.pdf) and (https://www.understandingsociety.ac.uk/sites/default/files/downloads/documentation/covid-19/questionnaires/wave-1/W1-covid-19-questionnaire.pdf), and the COVID Stress Scales (Taylor *et al.*, 2020). We included information about long-COVID and vaccination in a revised version in autumn 2021 where selected questions from the questionnaire on long-COVID by Davis *et al.* (2021) were used.

We aimed to cover domains directly related to occupational risk of infection (social distancing, human contact, contact with COVID-19-infected persons, use of personal protective equipment and face covering). In addition, we included domains covering risk factors more generally (health and demographics, psychosocial risk factors, and lifestyle risk factors). Finally, we included domains covering the social, economic physical, and economic consequences of the pandemic. Where possible, we selected questionnaire items and instruments from existing questionnaire resources. Additionally, where appropriate, we added newly developed questions.

These domains and questionnaire items were compiled into a *general occupational COVID-19 questionnaire* (34 core questions, and around 50 additional optional questions) and a specific *occupational COVID-19 questionnaire* (short version 8 questions, and 36 additional optional questions). The general occupational COVID-19 core questions are included in Supplementary Material (available at *Annals of Work Exposures and Health* online). All sources and references are furthermore included in the freely assessable material from the following website: https://omeganetcohorts.eu/resources/covid19-and-omega/. A first version of the questionnaires were available early February 2021. A slightly extended second version of the questionnaires were available ultimo December 2021. The most important changes were inclusion of questions on vaccinations and long-COVID. Drafts of the questionnaires were distributed to all participants in OMEGA-NET twice with >300 participants from 40 countries. We asked the OMEGA-NET participants to provide input on whether the questionnaires captured all the important occupational COVID-19-related information, and whether the questions were easy to understand and captured the proper information intended.

*The general occupational COVID-19 questionnaire* is a short core questionnaire covering key working life aspects of the COVID-19 pandemic described in the following domains:

- COVID-19 diagnosis and prevention (including vaccination)
- Health and demographics
- Use of personal protective equipment and face covering
- Health effects/work-related effects (including long-COVID)
- Financial effects
- Work-based risk factors
- Psychosocial risk factors
- Lifestyle risk factors
- Personal evaluation of the impact of COVID-19.

For each domain, additional questions are available in an extended version of the questionnaire.

The specific *occupational COVID-19 questionnaire* focusses on occupational risk factors for SARS-CoV-2 infection and COVID-19 disease and includes questions about:

- Type of job
- Amount of home working
- Social distancing
- Human contact (colleagues, patients, citizens)
- Commuting
- Use of personal protective equipment and face covering

Recently, Oude Hengel *et al.* (2021) developed a COVID-19 job exposure matrix based on similar occupational risk factors and mitigating factors as included in the occupational COVID-19 questionnaire. The COVID-19 job exposure matrix is also available on the OMEGA-NET homepage.

## Discussion

This initiative aims to encourage the use of similar working life core questions on COVID-19 across epidemiological studies within and between countries, and might facilitate any future data pooling or meta-analysis initiatives. The questionnaires have already frequently been downloaded (by April 2022, 904 times for the general occupational COVID-19 questionnaire and 514 times for the specific occupational COVID-19 questionnaire) from the OMEGA-NET (omeganetcohorts.eu), and the EPHOR [EPHOR—EPHOR Project (ephor-project.eu)]. homepages. Some of the questions are currently being used in the EPHOR project. More updates of the questionnaires are planned in order to meet future needs. For example causes of people changing or losing jobs may be related to the pandemic; this is not well covered in the present second version. In addition, more detailed information on vaccinations and reinfections will need to be included.

One strength of the questionnaires is the broad approach to various important working life issues related to SARS-CoV-2 infection, COVID-19 disease, and potentially future pandemics. The comprehensive approach to specific occupational risk factors is another strength. It requires more work to validate the questionnaires further, and we welcome collaboration with researchers willing to do this.

A limitation is the moderate number of questions for each of the domains in the general occupational questionnaire. Additionally, the numbers of questions on general core information such as ethnicity, demographics, lifestyle factors, and general health status are limited. We aimed at providing an instrument focussing on questions related to the SARS-CoV-2 pandemic. The questionnaires can be integrated in existing questionnaires already covering sociodemographic and health-related aspects. Examples are the abundantly used European Community Respiratory Health Survey ECRHS questionnaires on respiratory health and lifestyle factors (https://www.ecrhs.org/questionnaires-and-protocols) and the perceived stress scale questionnaire (https://www.das.nh.gov/wellness/Docs/Percieved%20Stress%20Scale.pdf).

Finally, it has to be mentioned that some issues related to specific occupations (e.g. seafarers), are not covered by the questionnaires.

We suggest that questions with similar content should be used in a sequence and the content and the order of the questions over different study waves (e.g. within and after the pandemic) should be the same to allow for appropriate comparisons over time. It is important to recognize that the questionnaires will need to be reviewed and revised depending on the stage of the pandemic (e.g. vaccination status, possible changes in pathogenicity, and symptom profiles from new variants), and the questionnaires also need to be adopted to potential future use when COVID has become endemic, or we may face future new pandemics. Country and cultural-specific adaptations might also be needed.

## Supplementary Material

wxac044_suppl_Supplementary_Material

## Data Availability

No data were used in this study.

## References

[CIT0001] Beale S , Patel P, Rodger A et al (2022) Occupation, work-related contact, and SARS-CoV-2 anti-nucleocapsid serological status: findings from the virus watch prospective cohort study. Occup Environ Med. doi:10.1136/oemed-2021-107920 PMC9072780 35450951

[CIT0002] Davis HE , Assaf GS, McCorkell L et al (2021) Characterizing long COVID in an international cohort: 7 months of symptoms and their impact. EClinicalMedicine; 38:101019. doi:10.1016/j.eclinm.2021.101019 34308300 PMC8280690

[CIT0003] Froessl LJ , Abdeen Y. (2021) The silent pandemic: the psychological burden on frontline healthcare workers during COVID-19. Psychiatry J; 2021:2906785. doi:10.1155/2021/2906785 34631873 PMC8497139

[CIT0004] Iavicoli S , Boccuni F, Buresti G et al (2021) Risk assessment at work and prevention strategies on COVID-19 in Italy. PLoS One; 16:e0248874. doi:10.1371/journal.pone.0248874 33740016 PMC7978285

[CIT0005] Lan FY , Wei CF, Hsu YT et al (2020) Work-related COVID-19 transmission in six Asian countries/areas: a follow-up study. PLoS One; 15:e0233588. doi:10.1371/journal.pone.0233588 32428031 PMC7237000

[CIT0006] Magnusson K , Nygård K, Methi F et al (2020) Occupational risk of COVID-19 in the first versus second epidemic wave in Norway, 2020. Euro Surveill; 26:2001875. doi:10.2807/1560-7917.ES.2021.26.40.2001875 PMC8511752 34622761

[CIT0007] Mutambudzi M , Niedwiedz C, Macdonald EB et al (2020) Occupation and risk of severe COVID-19: prospective cohort study of 120 075 UK Biobank participants. Occup Environ Med; 78: 307–14. 10.1136/oemed-2020-106731 PMC7611715 33298533

[CIT0008] Mylle G , Vanacker H, Verbeek C et al (2021) Prevalence of SARS-CoV-2 among Belgian workers in long-term care facilities. Occup Med (Lond); 71: 290–3. 34165551 10.1093/occmed/kqab076 PMC8344739

[CIT0009] Nafilyan V , Pawelek P, Ayoubkhani D et al (2021) Occupation and COVID-19 mortality in England: a national linked data study of 14.3 million adults, medRxiv, doi:10.1101/2021.05.12.21257123, preprint: not peer reviewed. 34965981

[CIT0010] Nelson TL , Fosdick BK, Biela LM et al (2021) Association between COVID-19 exposure and self-reported compliance with public health guidelines among essential employees at an institution of higher education in the US. JAMA Netw Open; 4:e2116543. doi:10.1001/jamanetworkopen.2021.16543 34287634 PMC8295736

[CIT0011] Oude Hengel KM , Burdorf A, Pronk A et al (2021) Exposure to a SARS-CoV-2 infection at work: development of an international job exposure matrix (COVID-19-JEM). Scand J Work Environ Health; 48: 61–70. 34788471 10.5271/sjweh.3998 PMC8729167

[CIT0012] Pauwels S , Boets I, Polli A et al (2021) Return to work after long COVID: evidence at 8th March 2021. Report HSE (Health and Safety Executive).

[CIT0013] Pearce N , Rhodes S, Stocking K et al (2021) Occupational differences in COVID-19 incidence, severity, and mortality in the United Kingdom: available data and framework for analyses. Wellcome Open Res; 6:102. doi:10.12688/wellcomeopenres.16729.1 34141900 PMC8188261

[CIT0014] Pronk A , Loh M, Kuijpers E et al; EPHOR Consortium. (2022) Applying the exposome concept to working life health: the EU EPHOR project. Environ Epidemiol; 6: e185. 35434456 10.1097/EE9.0000000000000185 PMC9005258

[CIT0015] Rayner C , Campbell R. (2021) Long Covid implications for the workplace. Occup Med (Lond); 71: 121–3. 33822179 10.1093/occmed/kqab042

[CIT0016] Senia P , Vella F, Mucci N et al (2022) Survey on COVID-19-related mortality associated with occupational infection during the first phase of the pandemic: a systematic review. Exp Ther Med; 23:10. doi:10.3892/etm.2021.10932 34815762 PMC8593876

[CIT0017] Taylor S , Landry CA, Paluszek MM et al (2020) Development and initial validation of the COVID Stress Scales. J Anxiety Disord; 72: 102232. 32408047 10.1016/j.janxdis.2020.102232 PMC7198206

[CIT0018] Turner MC , Mehlum IS. (2018) Greater coordination and harmonisation of European occupational cohorts is needed. Occup Environ Med; 75: 474–6. 29735748 10.1136/oemed-2017-104955 PMC6035485

[CIT0019] Van der Molen HF , Kezic S, Visser S et al (2020) Occupational COVID-19: what can be learned from notifications of occupational diseases? Occup Environ Med; 78: 464. 10.1136/oemed-2020-107121 PMC8142446 33158969

[CIT0020] Verbeeck J , Vandersmissen G, Peeters J et al (2021) Confirmed COVID-19 cases per economic activity during autumn wave in Belgium. Int J Environ Res Public Health; 18:12489. doi:10.3390/ijerph182312489 34886215 PMC8656663

[CIT0021] Würtz EML , Kinnerup MB, Pugdahl K et al (2022) Healthcare workers’ SARS-CoV-2 infection rates during the second wave of the pandemic: prospective cohort study. Scand J Work Environ Health. In press. 10.5271/sjweh.4049 PMC10539104 35780381

