# Supplementary material for: Development of Harmonized COVID-19 Occupational Questionnaires
Source: Ann Work Expo Health. 2022 Jul 10;67(1):4–8. doi: 10.1093/annweh/wxac044 (PMC9278210; doi:10.1093/annweh/wxac044)
Supplement: wxac044_suppl_Supplementary_Material [file wxac044_suppl_supplementary_material.pdf]

**Supplementary material. OMEGA-NET general occupational COVID-19 core questions**

**Title:**

Development of harmonised COVID-19 occupational questionnaires

**Authors:**

Vivi Schlünssen[1,2], Jean Baptist du Prel[3], Martie van Tongeren[4], Lode Godderis[5,6], Michelle C Turner [7-9], Damien McElvenny[4,10] on behalf of the OMEGA-NET COVID-19 Questionnaire Subtask Group

1 Department of Public Health, Work, Environment and Health, Danish Ramazzini Centre, Aarhus University, Aarhus, Denmark

2 National Research Center for the Working environment, Copenhagen, Denmark

3 Department of Occupational Health Science, University of Wuppertal, Germany

4 Centre for Occupational and Environmental Health, School or Health Sciences, University of Manchester, Manchester UK

5 Centre for Environment and Health, KU Leuven, Leuven, Belgium

6 IDEWE, External Service for Prevention and Protection at Work, Heverlee, Belgium

7 Barcelona Institute for Global Health (ISGlobal), Barcelona, Spain

8 Universitat Pompeu Fabra (UPF), Barcelona, Spain

9 CIBER Epidemiología y Salud Pública (CIBERESP), Madrid, Spain

10 Research Group, Institute of Occupational Medicine, Edinburgh, UK

**Supplementary material. OMEGA-NET general occupational COVID-19 core questions**

The questions are in general aiming at the current situation in relation to the COVID-19 pandemic. Questions will need to be modified depending on the nature of the study and the time period of interest, e.g. during early lockdown, following easing of lock down etc.

## Questions

### COVID-19 diagnosis & prevention

Have you had one or more of the following symptoms (YES/NO)? (Running or blocked nose - Cough - Sore throat - Dyspnea - Headache - Muscle ache/pain - Fever - Diarrhea or stomach pain - Reduced or lost sense of taste and smell - Unusual tiredness - Chest pain - Skin rashes - Problems with concentration or memory problems - Sadness or despair)
Have you been diagnosed with COVID-l9 on the basis of a test by a doctor/general practitioner /others? (YES/NO)
If yes - please state date of diagnosis: ________________
Have you ever been hospitalised due to COVID-l9? (YES/NO)
Have you been vaccinated for COVID-19? (YES/NO) If yes - Date of 1. vaccination___________ Date of 2. vaccinaton _____________ Date of 3. vaccinaton _____________

### Health

Do you suffer from a chronic disease or a chronic condition? (YES/NO)
Are you pregnant ? (YES/NO)

### PPE

Do you use any personal protective equipment during your work?

| | |
|---|---|
| a. Respiratory protective equipment | ALL DAY/MOST OF THE DAY/OCCASIONALLY |
| b. Face fit tested? | YES/NO/DON'T KNOW |
| c. Face covering (home made or surgical mask) | ALL DAY/MOST OF THE DAY/OCCASIONALLY |
| d. Gloves | ALL DAY/MOST OF THE DAY/OCCASIONALLY |
| e. Visor | ALL DAY/MOST OF THE DAY/OCCASIONALLY |
| f. Other, state what _______________________ | ALL DAY/MOST OF THE DAY/OCCASIONALLY |

### Health effects

Has the COVID-19 pandemic directly or indirectly worsened an existing disease/condition (YES/NO)
Has the COVID-19 pandemic directly or indirectly initiated negative health effects? (YES/NO)
Have you experienced issues with BRAIN FOG (inability to focus, think clearly, plan, process, understand, and maintain a coherent stream of thought; abnormally slow or fast thoughts) since the start of your COVID-19 illness? (YES/NO)
During your COVID-19 recovery, have you experienced any worsening or relapse of your symptoms after physical activity or mental activity? (YES/NO)

### Financial effects

How would you rate the changes of your personal financial situation before and after the beginning of the coronavirus crisis? (SIGNIFICANT WORSENING/SOMEWHAT WORSENING/SOMEWHAT IMPROVING/SIGNIFICANT IMPROVEMENT/NOT APPLICABLE)

### Work-based risk factors

Are you able to implement physical distancing (> 1/ 1.5 / 2 m) with your colleagues at all the time? (YES/NO)
Are you able to maintain physical distancing during commuting (above 1-2 m) (YES/NO)
How often does your current job require that you been potentially exposed to diseases or infections? (NEVER, SOMETIMES, OFTEN, FREQUENTLY, ALL OF THE TIME)
Has your job changed since the start of the pandemic? (YES/NO). What is your new job?

Is your main place of work, one of the following (YES/NO) (Home, Outdoor public place, Outdoors restricted (may need to clarify this) Hospital, Public transport vehicles, Public transport building and other public building with high throughput of public, Public buildings with low throughput, Shops and retail (public facing), Education, Non-public offices/manufacturing, Non-public transport).
Which of the following control measures are in place where you work (YES/NO) (Testing of staff/patients/public, Social distancing, Ventilation, Barriers partial or complete, PPE, Intensified cleaning)

What is the estimated number employed at the workplace in your current job?

### Psychosocial risk factors

I am worried about catching the virus (NOT AT ALL/RARELY/SOMETIMES/OFTEN/ALMOST ALWAYS)
I am worried about the effectiveness of social distancing (NOT AT ALL/RARELY/SOMETIMES/OFTEN/ALMOST ALWAYS)
I am worried that I can't keep my family safe from the virus (NOT AT ALL/RARELY/SOMETIMES/OFTEN/ALMOST ALWAYS)
Over the past 14 days I have felt burdened by media images of or news reports about COVID-19. (NOT AT ALL/RARELY/SOMETIMES/OFTEN/ALMOST ALWAYS)
Because of the COVID-19 pandemic, over the past 14 days I have felt stressed or burdened a lot by the curfew (NOT AT ALL/RARELY/SOMETIMES/OFTEN/ALMOST ALWAYS)
In the last 4 weeks, how often did you feel lonely? (HARDLY EVER OR NEVER/SOME OF THE TIME/OFTEN)

### Lifestyle factors

Are you a smoker (this applies even if you only smoke the odd cigarette/cigar or pipe every week)? (YES/NO)
Thinking about last week, were you or others in your household able to eat healthy and nutritious food? (YES/NO)

How much time in total (at work AND leisure time) did you spend over the last 7 days doing vigorous physical activities?_________________HOURS/WEEK

Over the past 14 days I have consumed substantially more alcohol than usual (0= NOT AT ALL to 4 = VERY MUCH)

Over the past 14 days I have consumed considerably more drugs (e.g. tranquilizers, sleeping pills or stimulants) than usual (0 = NOT AT ALL to 4 = VERY MUCH)

## Personal evaluation of the impact of the Coronavirus

How would you rate the changes in your working conditions before and after the beginning of the corona crisis? (SIGNIFICANT WORSENING/SOMEWHAT WORSENING/SOMEWHAT IMPROVING/SIGNIFICANT IMPROVEMENT/NOT APPLICABLE)

How would you rate the changes in your employment before and after the beginning of the CORONA crisis? (SIGNIFICANT WORSENING/SOMEWHAT WORSENING/SOMEWHAT IMPROVING/SIGNIFICANT IMPROVEMENT/NOT APPLICABLE)

How would you rank the changes in your private life during the coronoavirus crisis? (SIGNIFICANT WORSENING/SOMEWHAT WORSENING/SOMEWHAT IMPROVING/SIGNIFICANT IMPROVEMENT/NOT APPLICABLE)

To what extent have you perceived the coronavirus pandemic as a threat to yourself? (NO THREAT AT ALL TO MYSELF (0) to EXTREME THREAT TO MYSELF (100))
